# Fe_3_O_4_@N‐Doped Interconnected Hierarchical Porous Carbon and Its 3D Integrated Electrode for Oxygen Reduction in Acidic Media

**DOI:** 10.1002/advs.202000407

**Published:** 2020-05-27

**Authors:** Yi Wang, Mingmei Wu, Kun Wang, Junwei Chen, Tongwen Yu, Shuqin Song

**Affiliations:** ^1^ The Key Lab of Low‐Carbon Chemistry & Energy Conservation of Guangdong Province School of Chemical Engineering and Technology School of Materials Science and Engineering Sun Yat‐sen University Guangzhou 510275 China

**Keywords:** hierarchical porous carbon (HPC), non‐precious metal catalysts (NPM), oriented wholly integrated electrodes (ORR), oxygen reduction reaction

## Abstract

The rational design of electrode structure with catalysts adequately utilized is of vital importance for future fuel cells. Herein, a novel 3D oriented wholly integrated electrode comprising core–shell Fe_3_O_4_@N‐doped‐C (Fe_3_O_4_@NC) nanoparticles embedded into N‐doped ordered interconnected hierarchical porous carbon (denoted as Fe_3_O_4_@NC/NHPC) is developed for the oxygen reduction reaction (ORR). The as‐prepared catalyst possesses novel structure and efficient active sites. In rotating disk electrode measurements, the Fe_3_O_4_@NC/NHPC exhibits almost identical ORR electrocatalytic activity, superior durability, and much better methanol tolerance compared with the commercial Pt/C in acidic media. To the authors’ knowledge, this is among the best non‐precious‐metal ORR catalysts reported so far. Importantly, the Fe_3_O_4_@NC/NHPC is successfully in situ assembled onto carbon paper by the electrophoresis method to obtain a well‐designed 3D‐ordered electrode. With improved mass transfer and maximized active sites for ORR, the 3D‐oriented wholly integrated electrode shows superior performance to the one fabricated by the traditional method.

## Graphical abstract

A 3D oriented wholly integrated electrode is fabricated by the electrophoresis method based on a highly promising Fe‐N*_X_*‐C catalyst. Thanks to the rapid mass‐transfer pathway and adequate exposure of catalytic sites, the oxygen reduction reaction performance of the as‐obtained electrode outperforms the electrode fabricated by the traditional electrode preparation method.

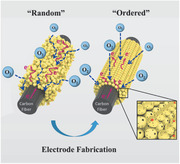

## 1 Introduction

Fuel cells are considered as one of the most efficient and clean energy conversion devices to meet the requirements for future energy‐demanding systems. The development of low‐cost, highly efficient, and stable non‐precious‐metal (NPM) catalysts, to replace the commonly used but rare and expensive Pt‐based catalysts, for oxygen reduction reaction (ORR) is critical to the wide‐spread implementation of fuel cells and has been a research focus in the field.^[^ ^1^ ^]^ Among various NPM catalysts, metal–nitrogen–carbon based materials (M‐N*_x_*/C, M = Fe, Co, Ni, etc.) have increasingly attracted researchers' interest and have been intensively pursued as one of the most promising candidates to replace Pt.^[^ ^2^ ^]^ Despite that much progress has been obtained, to date, the ORR catalytic performance of M‐N*_x_*/C is still insufficient compared with the commercial Pt catalysts, especially in the practical acidic media. The deep understanding of the intrinsic catalytic active sites of the M‐N*_x_*/C is of great importance for its further performance enhancement. The active sites of M‐N*_x_*/C are suggested to be the abundant nitrogen species, metal/metallic compounds, or the strong interaction between the M and N species, as reported in already published works.^[^ ^2^ ^]^ However, the real contribution of each ingredient is still under debate. Apart from that, ingeniously controlling the microstructure of the catalysts is another effective route to improve the performance of M‐N*_x_*/C catalyst.^[^ ^2^, ^3^ ^]^ Since the ORR is a multi‐phase reaction involving liquid, gas, and solid phases in fuel cells, a well‐designed microstructure could facilitate the ORR catalytic process in multiple ways: 1) providing rapid mass transfer path for ORR‐related species (O_2_, H^+^, OH^−^, and H_2_O); 2) affording sufficient surface area to maximize the exposure of accessible active sites; 3) protecting the catalyst nanoparticles from aggregation or dissolution, hence enhancing the long‐term durability.

Catalysts must be assembled into electrode when finally put in use. What is noteworthy is that, in the electrode, the available numbers of catalytic active sites not only rest with the catalyst materials themselves, but also strongly depend on the final microstructure of the electrode.^[^ ^4^ ^]^ In the traditional electrode fabrication process, the catalyst slurry, obtained by the mixture of the catalysts, ionomers (proton carriers), and solvent, is dropped or sprayed onto the gas diffusion layer (GDL). Undoubtedly, this will lead to disordered stacking structures with restricted mass transfer pathways and decreased active sites exposure. In this regard, ordering the microstructure of the electrode to gain an unimpeded ORR catalytic process is critical for improving the performance of fuel cells. Several attempts have been made by carefully designing the Pt‐based catalytic electrode, and successfully improved the performance of the electrode to some extent.^[^ ^5^ ^]^ Nevertheless, the high price and scarcity of Pt group metals significantly hinders the widespread commercialization of fuel cells.^[^ ^6^ ^]^ To our knowledge, the development of the ordered electrode based on M‐N*_x_*/C catalysts in fuel cells has rarely been reported.^[^ ^4f^ ^]^

Here, we report an excellent Fe‐N*_x_*/C catalyst with ordered interconnected hierarchical porous (HP) architecture and abundant catalytic sites. By introducing the precursor of Fe‐N*_x_*/C species, that is hemin, into the N‐doped HP carbon (NHPC) framework, the novel catalyst comprising Fe_3_O_4_@N‐doped‐C nanoparticles embedded in N‐doped ordered interconnected HP carbon (Fe_3_O_4_@NC/NHPC) is obtained. The important role of the NHPC framework in enhancing the ORR activity and the catalytic sites of Fe_3_O_4_@NC/NHPC is studied. The Fe_3_O_4_@NC/NHPC shows outstanding ORR activity and stability in acidic media. More interestingly, we develop a 3D‐oriented wholly integrated electrode by an electrophoresis method to in situ assemble the Fe_3_O_4_@NC/NHPC onto carbon paper (CP). The as‐prepared electrode outperforms the one fabricated by the traditional method in terms of ORR performance.

## 2 Results and Discussion

**Figure 1:**
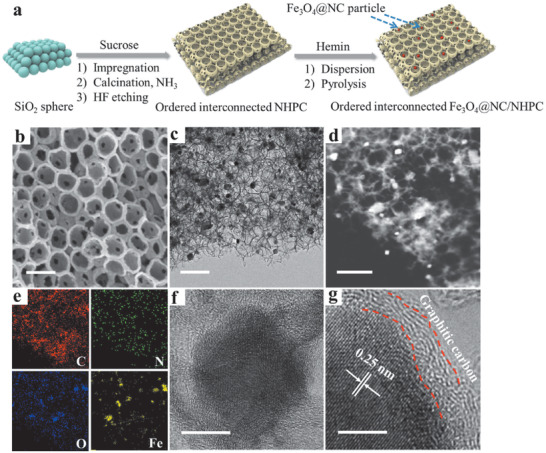
a) Synthetic protocol, b) SEM image, c) TEM image, d) STEM image, and e) the corresponding elemental mapping, f,g) HRTEM images of Fe_3_O_4_@NC/NHPC. Scale bar: b–d) 200 nm, f) 10 nm, g) 5 nm.

The fabrication of the Fe_3_O_4_@NC/NHPC was illustrated in **Figure** **1**a (for details see the Supporting Information). NHPC was firstly prepared through the polycondensation of sucrose into the SiO_2_ spheres array followed by the carbonization at 850 °C under flowing NH_3_ and HF etching (Figure S1, Supporting Information). The highly porous feature simultaneously with micro‐, meso‐, and macro‐pores was expected to be achieved at this stage. NH_3_ treatment is a facile and effective route for microstructure adjustment and its significant role is demonstrated in Supporting Information by comparing with the HP carbon carbonized under N_2_ (Figures S2–S8, Supporting Information). The as‐prepared NHPC was then subject to hemin (Fe and N source) in acetic acid and underwent a second heat treatment to introduce active ORR catalytic sites, and finally the Fe_3_O_4_@NC/NHPC was obtained. For comparison, another two catalysts, including NHPC without the hemin pyrolysis process and the sample directly pyrolyzed by hemin without NHPC (named Fe_3_O_4_@NC), were also prepared.

The morphology of the Fe_3_O_4_@NC/NHPC powder was investigated by field emission scanning electron microscopy (SEM), transmission electron microscopy (TEM), high‐resolution TEM (HRTEM), scanning TEM (STEM), and the corresponding elemental mappings. As shown in Figure 1b, Fe_3_O_4_@NC/NHPC consisted of a honeycomb‐like pore structure. Open hollow spherical macro‐pores were orderly arranged with an average diameter of ca. 150 nm and a wall thickness of 8–12 nm. The Fe_3_O_4_@NC/NHPC were interconnected through open windows with ≈10‐50 nm in diameter, providing favorable electron transfer property as well as open oxygen transfer pathways. The TEM image revealed that nanoparticles (NPs) with ca. 15 nm in size were well dispersed on the surface of hollow spherical macro‐pores (Figure 1c). Elemental mapping confirmed the existence of C, N, O, and Fe in Fe_3_O_4_@NC/NHPC with concentrated distribution of Fe and O on the high light sections of STEM and homogeneous distribution of C, N, O on the other sections (Figure 1d,e). HRTEM images revealed that NPs in Fe_3_O_4_@NC/NHPC had a core–shell structure with metal based NPs tightly encapsulated in the graphitic carbon shell, anchored to the NHPC substrate (Figure 1f,g). The crystalline lattices of the NPs could be easily identified by an interlayer spacing of 0.252 nm (Figure 1g), corresponding to the (311) plane of Fe_3_O_4_ (PDF card no. 65–3107), suggesting the existence of Fe_3_O_4_ in the sample.^[^ ^7^ ^]^ By contrast, the Fe_3_O_4_@NC directly pyrolyzed by hemin showed nearly no pore structure with metal‐based NPs hermetically wrapped in the thick carbon layer (Figure S9, Supporting Information).

**Figure 2:**
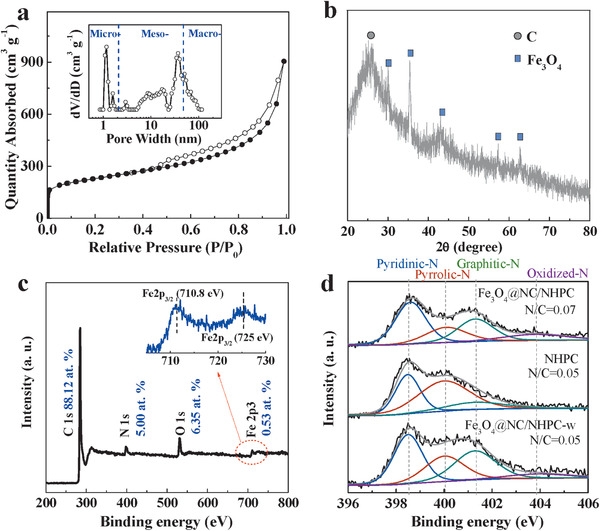
a) N_2_ adsorption‐desorption isotherms (inset is the corresponding pore size distribution curve), b) XRD spectrum, and c) XPS spectrum of Fe_3_O_4_@NC/NHPC. d) High‐resolution N1s XPS spectra of NHPC, Fe_3_O_4_@NC/NHPC, and Fe_3_O_4_@NC/NHPC‐w.

The porous nature of Fe_3_O_4_@NC/NHPC was further assessed by N_2_ adsorption–desorption analysis (**Figure** **2**a). The N_2_ adsorption–desorption isotherm exhibited the combined characteristics of type I/IV with a vertical rise at the initial region, a remarkable type H3 hysteresis loop at P/P_0_ of 0.45–1.0, and then a fast rise at high P/P_0_ region. This indicates the co‐existence of micro‐, meso‐, and macro‐pores in Fe_3_O_4_@NC/NHPC, which may lower the mass transfer resistance to the greatest extent. Generally, micro/mesopores can offer numerous active sites while meso/macropores can supply better mass diffusion and serve as an electrolyte reservoir.^[^ ^3^, ^8^ ^]^ DFT porosity distribution showed that there were micropores and a wide size range of meso‐ and macropores (2–100 nm), centered at 1.2 and 37.3 nm, respectively. The corresponding pore volume and BET surface area of Fe_3_O_4_@NC/NHPC were 1.40 cm^3^ g^−1^ and 768 m^2^ g^−1^, respectively. Such a hierarchical porosity and high specific surface area should be beneficial to the ORR catalytic process. The N_2_ adsorption‐desorption isotherm of Fe_3_O_4_@NC was also given in Figure S10, Supporting Information. The Fe_3_O_4_@NC primarily consisted of micro‐pores and a small number of mesopores. The pore volume and BET surface area of Fe_3_O_4_@NC were calculated to be 0.15 cm^3^ g^−1^ and 218 m^2^ g^−1^, respectively (Table S1, Supporting Information).

The bulk compositions of the Fe_3_O_4_@NC/NHPC were analyzed with X‐ray diffraction (XRD). As shown in Figure 2b, the XRD patterns consisted of an obvious peak at around 25°, corresponding to the diffraction from the (002) index of graphitic carbon. Five respective weak peaks at around 30.1, 35.4, 43.2, 57.2, and 62.8° indicate the formation of Fe_3_O_4_ (PDF card no. 65–3107). To gain further insight into the surface composition of Fe_3_O_4_@NC/NHPC, X‐ray photoelectron spectroscopy (XPS) was conducted and the survey spectra revealed that the corresponding surface atomic percentages were 88.12 at% of C, 6.35 at% of O, 5.00 at% of N, and 0.53 at% of Fe (Figure 2c). The Fe 2p spectrum showed characteristics of Fe_3_O_4_ with two peaks located at 710.8 and 725 eV, corresponding to the Fe 2p_3/2_ and 2p_1/2_ states, respectively (Figure 2c).^[^ ^9^ ^]^ The absence of the satellite peaks also corroborated the assignment of the final product to Fe_3_O_4_ rather than Fe_2_O_3_.^[^ ^10^ ^]^ The N1s spectrum could be fitted into four signals with 41.3 at% pyridinic N, 20.1 at% pyrrolic N, 26.2 at% graphitic N, and 12.3 at% oxidized N (Figure 2d).^[^ ^3d^ ^]^ Some research distribute an extra peak to be N‐Fe to declare the formation of N‐Fe interaction.^[^ ^9^, ^11^ ^]^ However, in the recent report of Fe‐N_x_/C catalysts, there were no N‐Fe signals separated from the XPS spectrum.^[^ ^12^ ^]^

**Figure 3:**
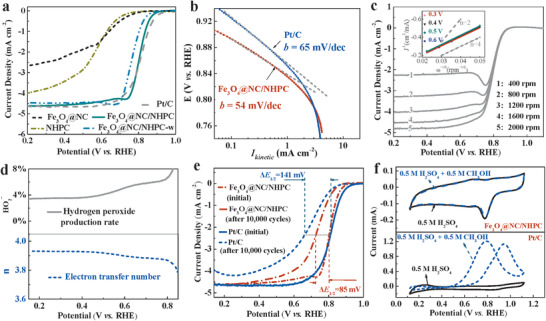
The ORR performance in O_2_‐saturated 0.5 m H_2_SO_4_ solution with a scan rate of 10 mV s^−1^ at room temperature. a) RDE voltammograms at 1600 rpm. b) Tafel plots obtained from the RDE data. c) RDE voltammograms of Fe_3_O_4_@NC/NHPC at various rotation speeds, and inset is the corresponding K–L plots. d) The electron transfer number (dotted line) and hydrogen peroxide production rate (solid line) of Fe_3_O_4_@NC/NHPC derived from RRDE data. e) RDE voltammograms before and after 10 000 cycles. f) CV curves in O_2_‐saturated 0.5 m H_2_SO_4_ solution with and without 0.5 m methanol.

The ORR activities of Fe_3_O_4_@NC/NHPC were evaluated in acidic media (0.5 m H_2_SO_4_) with the rotating disk electrode (RDE) measurements. For comparison, NHPC, Fe_3_O_4_@NC as well as a commercial Pt/C catalyst (40 wt% Pt, Johnson Matthey Corp.) were evaluated at the same experimental conditions. The cyclic voltammetry (CV) in N_2_‐saturated 0.5 m H_2_SO_4_ aqueous solution presented loop curves without the characteristic peaks for all samples except a small Fe redox peak for Fe_3_O_4_@NC (Figure S12, Supporting Information). In contrast, well‐defined cathodic peaks were observed in O_2_‐saturated electrolyte, suggesting the occurrence of ORR. It is worth noting that Fe_3_O_4_@NC shows an unusual, large explosion of electric current in the O_2_‐saturated electrolyte. This may originate from the intensive Fe redox in O_2_ due to unstable structure. As clearly observed in **Figure** **3**a, the linear sweep voltammogram (LSV) of Fe_3_O_4_@NC displayed much inferior ORR current density to Fe_3_O_4_@NC/NHPC, indicating the importance of the open surface and interconnected pore architecture for effective ORR catalytic process. The advantage of the ordered interconnected HP structure could be also seen from the relatively better ORR performance of NHPC than other common porous carbon materials (NOMC, NCNT, etc.) (Figure S13, Supporting Information). Besides, it could be found that the mass‐transfer‐free kinetic current density (*I*_kinetic_) at the potential of 0.80 V was much larger for Fe_3_O_4_@NC/NHPC (2.51 mA cm^−2^) than that for Fe_3_O_4_@NC (1.08 mA cm^−2^) (Figure S14, Supporting Information).

On the other hand, the Fe_3_O_4_@NC/NHPC exhibited a much higher ORR onset potential than that of NHPC, which could be ascribed to the more abundant active sites in Fe_3_O_4_@NC/NHPC (Figure 3a). In order to better understand the possible active sites, a concise study was conducted. i) Considering that the Fe_3_O_4_ particles may affect the catalyst activity, the sample with carbon‐coated Fe_3_O_4_ particles embedding in HPC (named as Fe_3_O_4_@C/HPC) was prepared to confirm its effect (Figures S15 and S16, Supporting Information). Unfortunately, Fe_3_O_4_@C/HPC possessed Fe_3_O_4_ particles but without N species and the possible N‐Fe interaction showed unsatisfactory ORR performance with a slightly higher ORR onset potential than HPC. This indicates that Fe_3_O_4_ particles make only very small contribution to the ORR process (Figure S17, Supporting Information). ii) N‐Fe interaction was commonly identified as the feature of Fe‐N_x_/C catalysts and considered to be critical for the good ORR performance of Fe‐N*_x_*/C catalysts.^[^ ^1^, ^12^ ^]^ Though there is no direct characterization for the N‐Fe interaction in our study, we cannot rule out its existence. To indirectly validate the possible N‐Fe interaction, Fe_3_O_4_@NC/NHPC was fully washed with concentrated acid to completely remove the Fe species, including the possible N‐Fe interaction. The resulted sample was marked as Fe_3_O_4_@NC/NHPC‐w (Figures S18–S20, Supporting Information). Fe_3_O_4_ particles were removed as well during this process; however, as discussed above, the effect of Fe_3_O_4_ particles can be almost ignored. Therefore, we can approximately attribute the difference of catalytic performance between Fe_3_O_4_@NC/NHPC‐w and Fe_3_O_4_@NC/NHPC to the lack of other Fe‐related species. As can be seen in Figure 3a, the onset potential of Fe_3_O_4_@NC/NHPC‐w was nearly 34 mV lower than that of Fe_3_O_4_@NC/NHPC, revealing the significant role of the active sites. As there are no other unknown Fe‐related active sites that arise in the characterization, it is suggested to be the common N‐Fe interaction. iii) As reported, N species played a vital role in determining the performance of N doping non‐metal ORR catalysts.^[^ ^13^ ^]^ The importance of N doping in our study can be primarily proved via the positive shift of ORR onset potential from HPC to NHPC. On the other hand, Fe_3_O_4_@NC/NHPC‐w, which possessed similar element composition and N content to NHPC, exhibited remarkable improvement in relation to NHPC in terms of ORR (Figures S21 and S22 and Table S2, Supporting Information). To gain a deeper insight into this phenomenon, the high‐resolution N 1s spectra of NHPC, Fe_3_O_4_@NC/NHPC, and Fe_3_O_4_@NC/NHPC‐w were carefully studied. As demonstrated in Figure 2d, the N configuration of Fe_3_O_4_@NC/NHPC‐w was almost identical to that of Fe_3_O_4_@NC/NHPC, but quite differed from that of NHPC (Table S3, Supporting Information). Graphitic‐N showed a distinct increment while the undesired pyrrole‐N decreased apparently in proportion from NHPC to Fe_3_O_4_@NC/NHPC‐w. The modification of N configuration might be caused by the introduction of hemin, and the following high‐temperature treatment with the existence of metal species thus made an impact on the ORR performance. These results indeed revealed that N doping with optimized configuration could greatly enhance the ORR performance of catalysts.

Compared with the commercial Pt/C catalyst, the Fe_3_O_4_@NC/NHPC exhibited an inspiring ORR activity with close ORR onset potential (0.898 V for Fe_3_O_4_@NC/NHPC *vs* 0.934 V for Pt/C) and equal half‐wave potential (*ca*. 0.803 V) as well as the same current density (*ca*. 4.77 mA cm^−2^) (Figure 3a). The excellent ORR activity of Fe_3_O_4_@NC/NHPC was also gleaned from a much smaller Tafel slope of 54 mV decade^−1^ than that measured with Pt/C (65 mV decade^−1^) (Figure 3b). To the best of our knowledge, this is among the best reported activities of various types of nonprecious catalysts in acidic media.

For further insight into the ORR kinetics of Fe_3_O_4_@NC/NHPC, a series of LSV curves at different rotating speeds were recorded and analyzed with Koutecky–Levich (K–L) equation (Figure 3c). The linearity of the K–L plots and the essentially coincident fitting lines suggested the first‐order reaction kinetics toward the concentration of dissolved O_2_ and identical electron transfer process for ORR at different potentials. Rotating ring disk electrode (RRDE) measurement gave an average electron transfer number (*n*) of 3.91 with HO_2_^−^ yields constantly below 6% at all investigated potentials (Figure 3d; Figure S23, Supporting Information). That was to say, the ORR catalyzed by Fe_3_O_4_@NC/NHPC was mainly the desired four‐electron process. The stability of Fe_3_O_4_@NC/NHPC was evaluated using an accelerated durability test protocol. As shown in Figure 3e, the ORR half‐wave potential for Fe_3_O_4_@NC/NHPC after 10 000 potential cycles was negatively shifted by 94 mV, while Pt/C showed a more negative shift of 141 mV. Although the initial ORR activity of Fe_3_O_4_@NC/NHPC was lower than that of Pt/C, it exhibited a better ORR performance than Pt/C after stability test (∆*E*_1/2_ = 51 mV). Conversely, Fe_3_O_4_@NC without NHPC framework was subjected to a huge performance decay after 10 000 potential cycles (∆*E*_1/2_ = 339 mV, Figure S24, Supporting Information). Besides, Fe_3_O_4_@NC/NHPC showed excellent methanol tolerance, while the Pt/C catalyst suffered a remarkably harsh methanol oxidation process (Figure 3f).

Based on the above analysis, we could draw a conclusion that a highly promising Fe‐N*_X_*/C catalyst possessing effective active sites was successfully prepared with the existence of HP architecture. To carry forward the outstanding performance of Fe_3_O_4_@NC/NHPC for electrode, that is, to keep the interconnected hierarchical pore system and abundant accessible catalytic sites during the electrode fabrication process, a 3D oriented wholly integrated electrode based on Fe_3_O_4_@NC/NHPC (Fe_3_O_4_@NC/NHPC/CP‐E) was then in situ fabricated by an electrophoresis process with the Toray carbon paper (CP) as the substrate.

**Figure 4:**
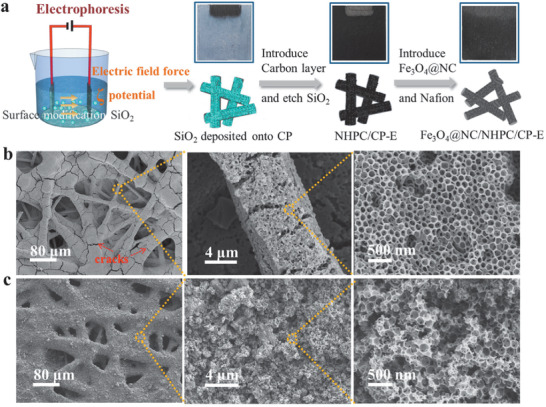
a) Fabrication process of Fe_3_O_4_@NC/NHPC/CP‐E. b) SEM images of Fe_3_O_4_@NC/NHPC/CP‐E at different magnification. c) SEM images of Fe_3_O_4_@NC/NHPC/GDL‐S at different magnification.

The fabrication process of the Fe_3_O_4_@NC/NHPC/CP‐E and the corresponding optical photographs are shown in **Figure** **4**. The fabrication method was inspired by a freestanding electrode in lithium–air batteries.^[^ ^14^ ^]^ Original CP was used as the substrate without any pre‐treatment. Firstly, SiO_2_ nanoparticles with regular particle size were deposited onto CP by an electrophoresis method, in which the SiO_2_ colloidal particles with an enhanced *ζ* potential moved directionally under an electric field force and finally deposited on the fibers of CP. Then, the template electrode was subjected to the same synthetic route as Fe_3_O_4_@NC/NHPC powder to obtain a honeycomb‐like Fe_3_O_4_@NC/NHPC/CP‐E electrode (Figure S25, Supporting Information). Finally, the electrode was treated by a dilute Nafion solution to both introduce proton conductor and optimize hydrophilic property. For comparison, the electrodes fabricated by a conventional spray method, spraying the Fe_3_O_4_@NC/NHPC catalysts ink onto PTFE treated CP, were prepared and denoted as Fe_3_O_4_@NC/NHPC/GDL‐S. The total catalyst loading for all samples was kept to be as equal as possible, that is, 0.23 mg cm^−2^ for Fe_3_O_4_@NC/NHPC/CP‐E, and 0.29 mg cm^−2^ for Fe_3_O_4_@NC/NHPC/GDL‐S. Meanwhile, the hydrophobic property of Fe_3_O_4_@NC/NHPC/CP‐E was similar to the referenced electrode as revealed by the contact angle tests (Figure S26, Supporting Information). That is to say, the Fe_3_O_4_@NC/NHPC/CP‐E possessed certain water management ability.

The morphology of Fe_3_O_4_@NC/NHPC/CP‐E was investigated by SEM at different magnifications (Figure 4b). As shown in the low‐magnification image, some of Fe_3_O_4_@NC/NHPC catalysts were anchored to CP along carbon fibers while the others were uniformly embedded in the space between fibers skeletons, leaving plenty of capacious interspace of the original CP for O_2_ bubbling. Besides, obvious cracks with ca. 1–3 µm in width were observed. These micropores were hardly flooded by liquid water and hence would favor the flow of reactants to neighboring catalytic sites.^[^ ^15^ ^]^ A closer observation of Fe_3_O_4_@NC/NHPC/CP‐E showed a continuously regular arrangement of interconnected HP carbons that were well anchored to the CP substrate. TEM, XPS, and N_2_ adsorption–desorption results of Fe_3_O_4_@NC/NHPC/CP‐E electrode were similar to those of the Fe_3_O_4_@NC/NHPC powder, revealing that related active catalytic sites and intact pore structure were successfully in situ constructed in the electrode (Figures S27–S30, Supporting Information). The as‐obtained 3D‐oriented wholly integrated electrode possessed many desired properties: 1) the interconnected hierarchical pore system for rapid ORR‐related species diffusion throughout the entire electrode; 2) highly open interfacial area for active sites exposure; 3) well‐regulated electron/proton transfer channels where the consecutive embedded NHPC matrix acted as high flux electron conductor and the Nafion network over the active sites acted as proton conductor. In contrast, in the case of CP, it was covered by heavy Fe_3_O_4_@NC/NHPC and PTFE without any crack in the Fe_3_O_4_@NC/NHPC/GDL‐S electrode (Figure 4c). The enlarged images presented a ruleless package of Fe_3_O_4_@NC/NHPC with unmatched pores and randomly distributed proton carriers, which would certainly increase the mass transfer pathways and decrease the amounts of accessible active sites.

**Figure 5:**
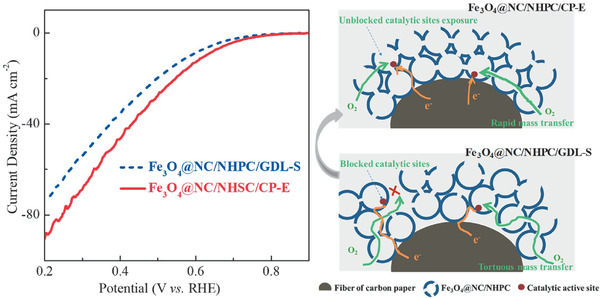
Oxygen reduction polarization curves of Fe_3_O_4_@NC/NHPC/GDL‐S (catalyst loading ca. 0.29 mg cm^−2^) and Fe_3_O_4_@NC/NHPC/CP‐E (catalyst loading ca. 0.23 mg cm^−2^) in O_2_‐saturated 0.5 m H_2_SO_4_. On the right side are the schematic illustrations of the mass and electron transfer path of Fe_3_O_4_@NC/NHPC/GDL‐S and Fe_3_O_4_@NC/NHPC/CP‐E.

The steady‐state ORR performances on the as‐prepared electrodes (1.0 cm × 1.0 cm) were evaluated in a home‐made three‐electrode cell.^[^ ^16^ ^]^ The Fe_3_O_4_@NC/NHPC/CP‐E afforded an ORR current density of ca. 87 mA cm^−2^ at −0.2 V, exceeding that of the Fe_3_O_4_@NC/NHPC/GDL‐S (*ca*. 70 mA cm^−2^) (**Figure** **5**). These results were consistent with the above analyses that the 3D oriented wholly integrated architecture of Fe_3_O_4_@NC/NHPC/CP‐E with increased transfer pathways (H_2_O/O_2_, H^+^/e^−^) and reaction regions was able to improve its ORR performance. Meanwhile, the ORR currents of Fe_3_O_4_@NC/NHPC/CP‐E were eight times higher than that of NHPC/CP‐E (ca.7 mA cm^−2^), suggesting that synergetic active sites of Fe_3_O_4_@NC/NHPC were successfully established and worked in Fe_3_O_4_@NC/NHPC/CP‐E (Figure S31, Supporting Information). Overall, benefiting from highly efficient catalytic activity of Fe_3_O_4_@NC/NHPC and extraordinary mass transfer pathways of the 3D oriented wholly integrated electrode, the Fe_3_O_4_@NC/NHPC/CP‐E showed excellent steady‐state ORR activity. Increasing the loading of Fe_3_O_4_@NC/NHPC/CP‐E by adjusting the electrophoresis parameters may further improve its performance and this study is under way.

## 3 Conclusion

In summary, we have developed an outstanding Fe_3_O_4_@NC/NHPC catalyst for ORR. Thanks to the combination of rational pore structure and effectively catalytic sites, the Fe_3_O_4_@NC/NHPC exhibits comparable ORR catalytic activity but superior stability and methanol tolerance to commercial Pt/C catalyst in acidic media. The catalytic active sites are revealed to be N‐Fe interaction and optimized N species modified by metal species. More interestingly, a 3D‐oriented wholly integrated Fe_3_O_4_@NC/NHPC/CP‐E has been successfully in situ fabricated by the electrophoresis method, which possesses interconnected HP structure, large interface for catalytic sites, and high electron/proton conductivity. The Fe_3_O_4_@NC/NHPC/CP‐E electrode shows an enhanced ORR catalytic performance than the traditional electrode. This study not only provides a novel non‐precious metal catalyst or electrode for fuel cells but also provides a general method to construct effective nanostructure for M‐N*_x_*/C catalyst and opens up a brand new approach to prepare spatially ordered electrode in many next‐generation power devices. Due to various precursors to fabricate M‐N*_x_*/C catalyst, more NHPC‐supported catalysts and corresponding oriented wholly integrated electrode could be designed and expected to be more efficient for application.

## Conflict of Interest

The authors declare no conflict of interest.

## Supporting information

Supporting Information
