# Supplementary material for: Fe3O4@N‐Doped Interconnected Hierarchical Porous Carbon and Its 3D Integrated Electrode for Oxygen Reduction in Acidic Media
Source: Adv Sci (Weinh). 2020 May 27;7(14):2000407. doi: 10.1002/advs.202000407 (PMC7375250; doi:10.1002/advs.202000407)
Supplement: Supplementary file 1 — Supporting Information [file ADVS-7-2000407-s001.pdf]

Copyright WILEY-VCH Verlag GmbH & Co. KGaA, 69469 Weinheim, Germany, 2020.

Supporting Information

Fe_3O_4@N-doped Hierarchical Porous Carbon and Its 3D Integrated Electrode for Oxygen Reduction in Acidic Media

*Yi Wang, Mingmei Wu, Kun Wang, Junwei Chen, Tongwen Yu, Shuqin Song**

1. Experimental Section

1.1 Catalyst preparation

1.1.1 Synthesis of SiO_2 template. SiO_2 spheres with a diameter of ~150 nm were synthesized by a modified Stöber method.[1] Typically, 259.0 mL of ethanol was mixed with 10.5 mL of deionized (DI) water and 11.2 mL of ammonia (32 wt. %). Then, the mixture was stirred for at least 30 min, followed by quick addition of 11.2 mL of tetraethylorthosilicate (TEOS). The resultant solution was stirred for 18 h at room temperature and then centrifuged at 7,000 rpm for 5 min, followed by desiccation at 50°C.

1.1.2 Synthesis of NHPC powder. The as-prepared SiO_2 spheres (2.0 g) were soaked in a sucrose aqueous solution (20 wt. %) for 20 min under magnetic stirring. The excess solution was removed by filtration and then dried at 70°C under vacuum for 12 h. The as-obtained white sucrose/SiO_2 was carbonized under flowing NH_3 (flow rate: 50 mL min^{-1}, heat rate: 5°C min^{-1}) at 850°C for 3 h. The SiO_2 templates were etched out by 10 wt. % HF to obtain packed N-doped hollow spherical carbon (NHPC). For the synthesis of hollow spherical carbon (HPC) powder, the sucrose/SiO_2 was carbonized under flowing N_2 at the same conditions as NHPC.

1.1.3 Synthesis of Fe_3O_4@NC. Hemin ($C_{34}H_{32}ClN_4O_4Fe$, Aladdin) (16.0 mg) was dissolved in 150 mL of acetic acid by stirring. After fully dried, the powder was subjected to a pyrolysis at 800°C for 3 h under flowing N_2 atmosphere (flow rate: 50 mL min^{-1}, heat rate: 5°C min^{-1}).

1.1.4 Synthesis of Fe_3O_4@NC/NHPC powder. Hemin ($C_{34}H_{32}ClN_4O_4Fe$, Aladdin) (16.0 mg) was dissolved in 150 mL of acetic acid and mixed with 20.0 mg of NHPC by stirring and ultrasonic dispersion. The above solution was then slowly evaporated at 70°C under constant stirring and almost dry powder was further dried at 70°C overnight under vacuum. Finally, the as-obtained powder was subjected to a second heat treatment at 800°C for 3 h under flowing N_2 atmosphere (flow rate: 50 mL min^{-1}, heat rate: 5°C min^{-1}).

1.1.5 Synthesis of Fe_3O_4@C/HPC powder. Terephthalic acid ($C_8H_6O_4$, Aladdin) and ferric chloride ($FeCl_3$, Aladdin) was chosen as carbon source and iron source, respectively. The terephthalic acid and ferric chloride went through a hydrothermal progress corresponding to the common synthesis method of Fe-MIL-88 metal organic framworks (MOFs) with the existence of HPC. The detailed dosage was studied to make sure that Fe_3O_4@C/HPC possessed similar Fe content to Fe_3O_4@NC/NHPC. After filtration, washing and fully drying process, the powder was subjected to a pyrolysis at 800°C for 3 h under flowing N_2 atmosphere (flow rate: 50 mL min^{-1}, heat rate: 5°C min^{-1}).

1.1.6 Synthesis of Fe_3O_4@NC/NHPC-w powder. The as-prepared Fe_3O_4@NC/NHPC powder was dispersed in 1.0 mol/L H_2SO_4 and stirred for 24 h, followed by filtration.

1.1.7 Fabrication of SiO_2/CP. The SiO_2 deposited on the Toray carbon paper (SiO_2/CP) template was fabricated by a modified electrophoresis method.[2] The fabrication process involved two key steps: (1) surface functionalization of SiO_2 spheres, and (2) electrophoretic deposition. For the surface functionalization of SiO_2 spheres, 6.0 mL of mercaptopropyltrimethoxysilane (MPS, $C_6H_{16}O_3SSi$, Aladdin) was added to 200 mL of a dried toluene solution containing 2.0 g of SiO_2 spheres, and the resulting mixture was refluxed at 110°C for 12 h. The solution was cooled down to room temperature and filtered, washed with toluene, ether, acetone, and methanol, and then dried at 50°C. The

surface-modified SiO_2 spheres were referred to as MPS-SS. For the electrophoretic deposition, 100.0 mg of MPS-SS were dispersed into a mixed solvent of acetone and methanol (100 mL: 100 mL) after ultrasonication for 1 h. The electrophoretic deposition of the MPS-SS was performed in a 300 mL beaker with a vertical CP working and a CP counter cathode connected to a direct current power supply through a home-made electrode holder. The size of CP working and counter electrodes were 2.0 cm×2.0 cm and 2.4 cm×2.4 cm, respectively. These two electrodes were kept parallel at about 1.8 cm apart in the suspension. During the electrophoretic process, a constant deposition voltage of 25-30 V was used for the electrophoretic deposition for 15-20 min.

1.1.8 Fabrication of Fe_3O_4@NC/NHPC/CP-E electrode. The Fe_3O_4@NC/NHPC/CP-E electrode was in-situ fabricated using the same method as Fe_3O_4@NC/NHPC powder. Briefly, the obtained SiO_2/CP was soaked in a sucrose solution (20 wt. %) for 20 min, and then was put on a clear filter paper to dry at 70^oC under vacuum for 3 h. The as-obtained sample was carbonized under flowing NH_3 (flow rate: 50 mL min^{-1}, heat rate: 5^oC min^{-1}) at 850^oC for 3 h. The SiO_2 templates were etched out by 10 wt. % HF overnight. The loading of NHPC was ~0.20 mg cm^{-2}. Next, 4.0 mg of hemin was dispersed in 40.0 mL acetic acid in a flat bottom beaker. The NHPC/CP was placed on the bottom of the beaker and the solution was slowly evaporated at 70^oC statically to gain a uniform distribution of hemin on NHPC/CP. After dried at 70^oC overnight under vacuum, the as-obtained sample was finally subjected to a second heat treatment at 800^oC for 3 h under flowing N_2 atmosphere (flow rate: 50 mL min^{-1}, heat rate: 5^oC min^{-1}). The loading of Fe_3O_4@NC/NHPC was about 0.23 mg cm^{-2}. The as-prepared electrode was soaked into an extraordinary dilute Nafion solution (0.5 wt. %) for 2 min and then dried at 80^oC under vacuum for 12 h. The total mass loading of Fe_3O_4@NC/NHPC/CP-E was ~0.30 mg cm^{-2}.

1.1.9 Fabrication of Fe_3O_4@NC/NHPC/GDL-S electrode. Before the fabrication of electrode, a waterproofing treatment of original CP was conducted. The original CP was soaked into a 2.0 wt. % PTFE solution for dozens of seconds and then blow-dried and repeated to reach 30 wt. % PTFE in CP.

The above-treated CP was then subjected to annealing at 340 ^{o}C in air for 1 h and the gas diffusion layer (GDL) was achieved. For the fabrication of Fe_3O_4@NC/NHPC/GDL-S electrode, a catalyst slurry with 5.6 mg Fe_3O_4@NC/NHPC powder, 14.3 µL Nafion solution (5 wt. %, Dupont), 6.3 mL isopropanol was sprayed onto the as-prepared GDL (2.0 cm × 2.5 cm) and then dried at 80°C under vacuum for 12 h. The total mass loading was 0.32 mg cm^{-2} (10 % of Nafion to catalyst ratio) for Fe_3O_4@NC/NHPC/GDL-S electrode.

1.2 Materials characterization

Scanning electron microscopy (SEM) was performed on a Quanta 400FEG. Transmission electron microscopy (TEM) images, high-resolution TEM (HRTEM) images, scanning transmission electron microscopy (STEM) images and element mapping were acquired on a JEM-2010 (HR) operating at an accelerating voltage of 200 kV. Wide-angle X-ray diffraction (XRD) patterns were recorded on a D-MAX 2200 VPC diffractometer using Cu K radiation (40 kV, 26 mA). X-ray photoelectron spectroscopy (XPS) characterization was performed by an ESCALAB 250. N_2 adsorption-desorption analysis was conducted using micromeritics®. Before measurements, all samples were degassed at 300 ^{o}C for at least 4 h. The specific surface area was determined by the standard BET method in the relative pressure range of 0.05-0.3. The pore size distribution (PSD) curves were calculated by the nonlocal density functional theory (DFT).

1.3 Electrochemical measurements

1.3.1 Rotating disk electrode (RDE) measurements. For the as-prepared catalysts, 7.0 mg of catalyst sample was ultrasonically dispersed in the mixed solution of 350.0 µL of ethanol and 95.0 µL of Nafion® solution (5 wt. %) for 1 h, and 5.0 µL of ink (containing 78.5 µg of catalyst) was dropped onto a glassy carbon RDE of 5.0 mm in diameter (loading ~ 0.4 mg cm^{-2}). For the commercial Pt/C (20 wt. Pt%, Johnson Matthey Corp.), 5.0 mg of the samples was ultrasonically dispersed in the solution of 1.8 mL of ethanol and 0.2 mL of Nafion® solution for 1 h, and 12.0 µL of ink was dropped onto the glassy carbon RDE with a catalyst loading of 30 µg Pt cm^{-2}.

The electrochemical measurements were conducted on an Auto84480 instrument with a Pine Instruments in a three-electrode cell using a saturated calomel electrode (SCE) as the reference electrode, a Pt foil as the counter electrode and the sample modified glassy carbon electrode as the working electrode. All potentials in this study are referred to as that of the reversible hydrogen electrode (RHE). The potential difference between SCE and RHE is calculated based on Equation (1):

$$E(\text{RHE}) = E(\text{SCE}) + 0.0591\text{pH} + 0.24 \quad (1)$$

The electrolyte was saturated with O_2/N_2 by bubbling O_2/N_2 prior to the start of each experiment for at least 30 min. The scan rate of all measurements was kept constant at 10 mV s^{-1}. The working electrode was cycled at least 40 times in the N_2-saturated electrolyte before data were recorded. For Cyclic voltammetry (CV), no rotating speed was applied on the working electrode and the gas flow was stopped during the recording process in order to gain non-vibrating curves. For RDE measurements, the working electrode was scanned at varying rotating speeds from 400 to 2000 rpm with gas flow maintained. The background that arose from the remarkable ion adsorption on the surface of catalysts was eliminated by subtracting the current density value of the N_2-saturated measurement from that of the O_2-saturated measurement. Koutecky-Levich (K-L) plots were analyzed at various electrode potentials. For the Tafel plot, the kinetic current was calculated from the mass-transfer correction of RDE by: [3]

$$i_K = \frac{i \times i_L}{(i_L - i)} \quad (2)$$

where i is the measured current density, i_L and i_K are the diffusion-limiting and kinetic current densities, respectively.

The stability of catalysts were evaluated using an accelerated durability test protocol by potential cycling the catalyst between 0.6 and 1.0 V (vs. NHE) at 50 mV s^{-1} in O_2-saturated electrolyte.

1.3.2 Rotating ring-disk electrode (RRDE) measurements. Catalyst inks were prepared by the same method as RDE. The catalyst ink (6.3 µL, containing 98.9 µg of catalyst) was dropped onto a glassy

carbon rotating disk electrode of 5.6 mm in diameter (loading ~ 0.4 mg cm^{-2}). The ring current (I_R) was measured with a Pt ring electrode. Pt ring electrode was polarized at 1.2 V (*vs.* SCE) in the O_2-saturated 0.5 M H_2SO_4 solution. The four-electron selectivity of ORR on the catalyst was calculated based on Equation (3). The H_2O_2 yield was determined by Equation (4).[3]

$$n = \frac{4\times I_D}{I_D + \frac{I_R}{N}} \tag{3}$$

$$\% HO_2^- = \frac{4-n}{2} \tag{4}$$

where I_D is the disk current, I_R is the ring current, and N is H_2O_2 collection efficiency of the Pt ring with the value of 0.37.

1.3.3 ORR catalytic activities on carbon paper. ORR performance measurements based on the carbon paper made reference to ref. 3. The working electrode was prepared as stated above and cut into 1.0 cm × 2.0 cm. The measurements were conducted on an Auto84480 instrument in a home-made three-electrode cell using SCE as the reference electrode, a Pt foil as the counter electrode and the as-prepared electrode as the working electrode, respectively. The working electrode was immersed in the electrolyte. A flow of O_2/N_2 (40 mL min^{-1}) was maintained bubbling in the electrolyte during the measurements. The working electrode was cycled at least 40 times in N_2-saturated electrolyte before data were recorded at a scan rate of 10 mV s^{-1} for ORR measurements.

2. Supplementary Figures

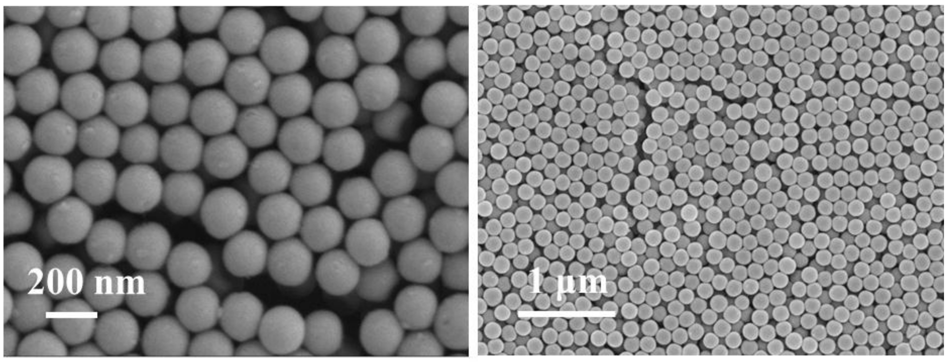

Figure S1. SEM images of SiO_2 sphere.

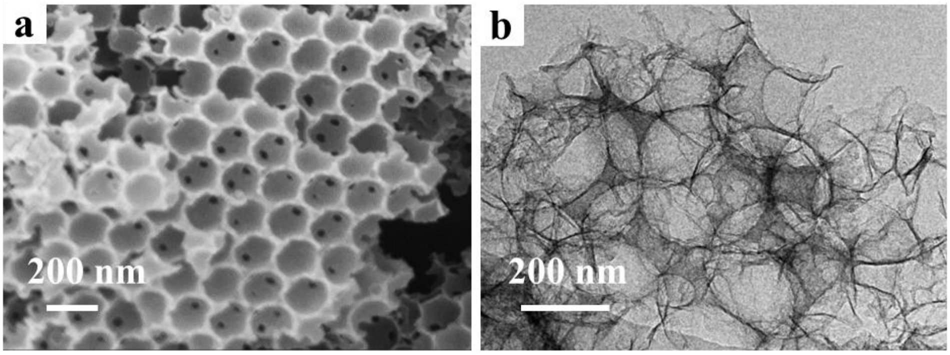

Figure S2. a) SEM image, b) TEM image of HPC.

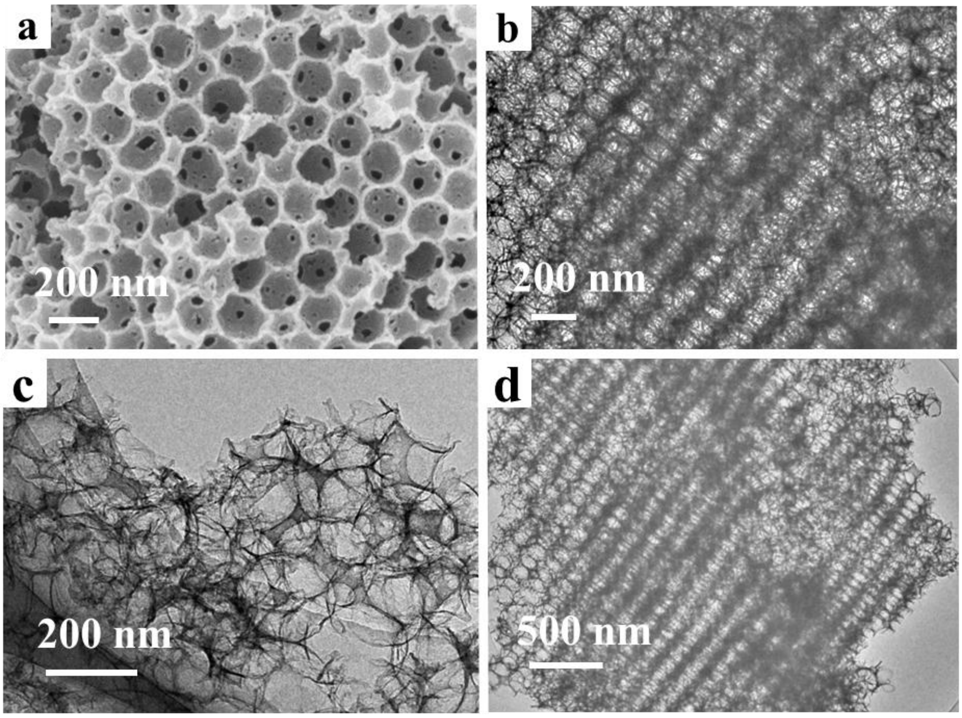

Figure S3. a) SEM image, b-d) TEM images of NHPC.

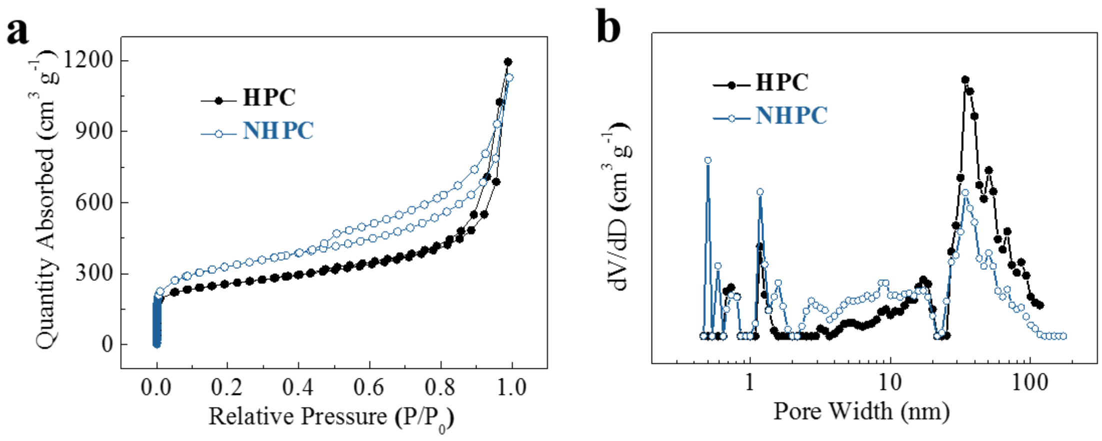

Figure S4. a) N_2 adsorption-desorption isotherms, b) the corresponding pore size distribution curves of HPC and NHPC.

NH_3 activation was an important pore modification method in the fabrication of Fe_3O_4@NC/NHPC. The N_2-sorption isotherm of HPC exhibited an inapparent capillary condensation in the middle-pressure range and a type H3 hysteresis loops, indicating dominated macropores and less small-mesopores existing in HPC. With the NH_3 activation, NHPC showed a clear characteristic for mesoporous materials, and the pore structure is well inherited by Fe_3O_4@NC/NHPC. Besides, the N-doping of carbon may do help in a stronger coupling between M and N-doped carbon. [4]

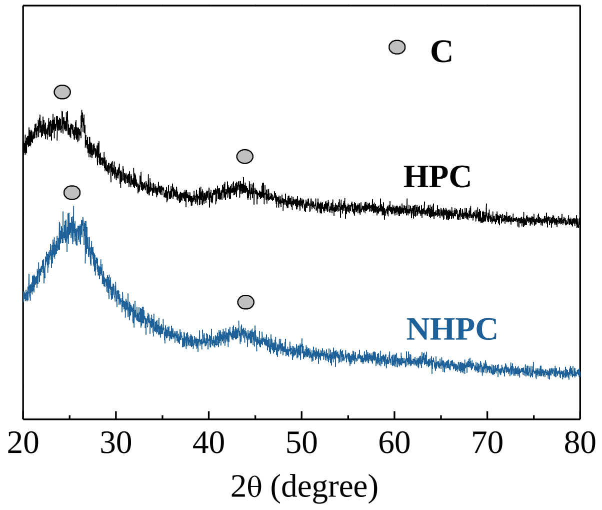

Figure S5. XRD spectra of HPC and NHPC.

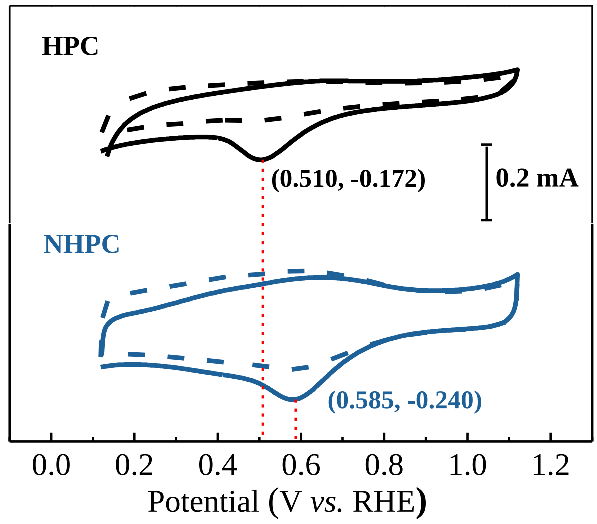

Figure S6. CV curves of HPC and NHPC in N_2-saturated 0.5 M H_2SO_4 solution (dash line) and O_2-saturated 0.5 M H_2SO_4 solution (solid line).

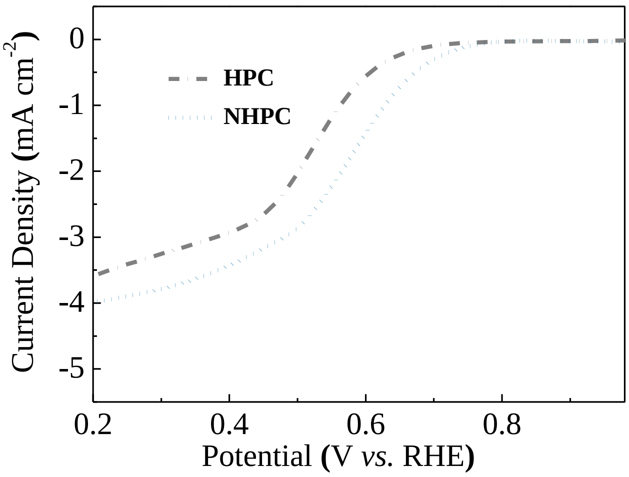

Figure S7. LSV curves for HPC and NHPC in O_2-saturated 0.5 M H_2SO_4 solution at 1600 rpm.

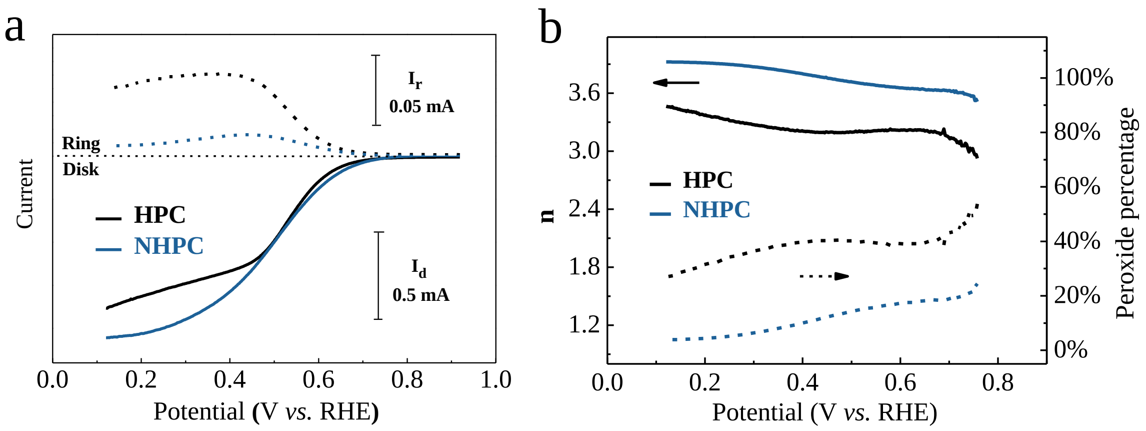

Figure S8. a) RRDE voltammograms recorded with HPC and NHPC in O_2-saturated 0.5 M H_2SO_4 solution at 1 600 r.p.m. The disk potential was scanned at 10 mV/s and the ring potential was constant at 1.2 V versus SCE. b) the electron transfer number (n) (solid line) and percentage of peroxide (dotted line) of HPC and NHPC based on the corresponding RRDE data in a.

The HO_2^- yields of HPC decreased with the potential negatively shift. The respective electron transfer number was 3.16-3.46 for HPC, 3.64-3.93 for NHPC in 0.5 M H_2SO_4. This suggested that pure hollow carbon was prone to a half 2-e and half 4-e ORR process. After nitrogen doping, which was one of the most effective strategies to create active sites in carbon materials, the NHPC was more likely to go through a 4-e ORR process

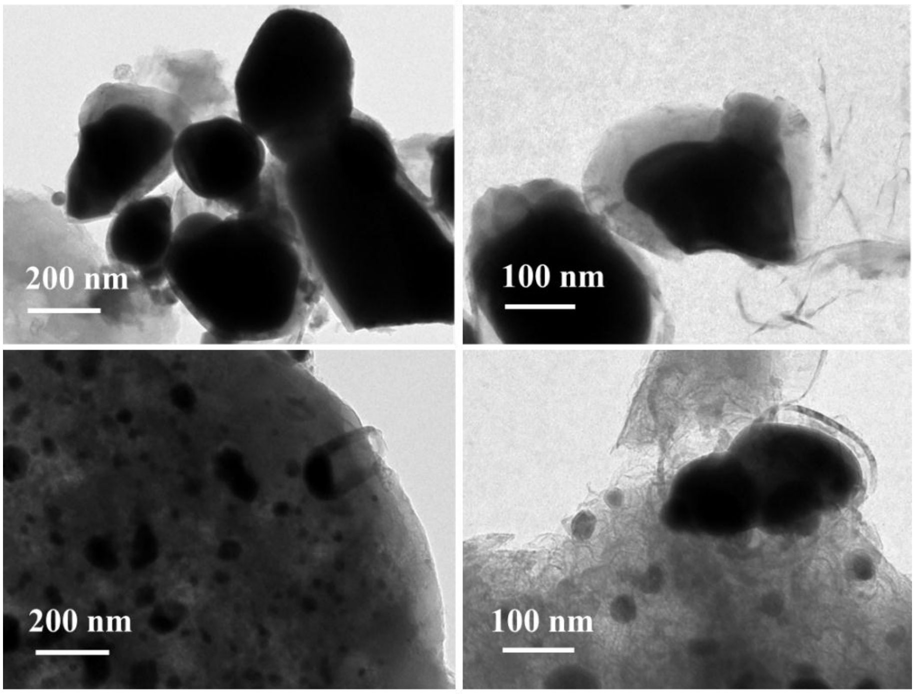

Figure S9. TEM images of Fe_3O_4@NC.

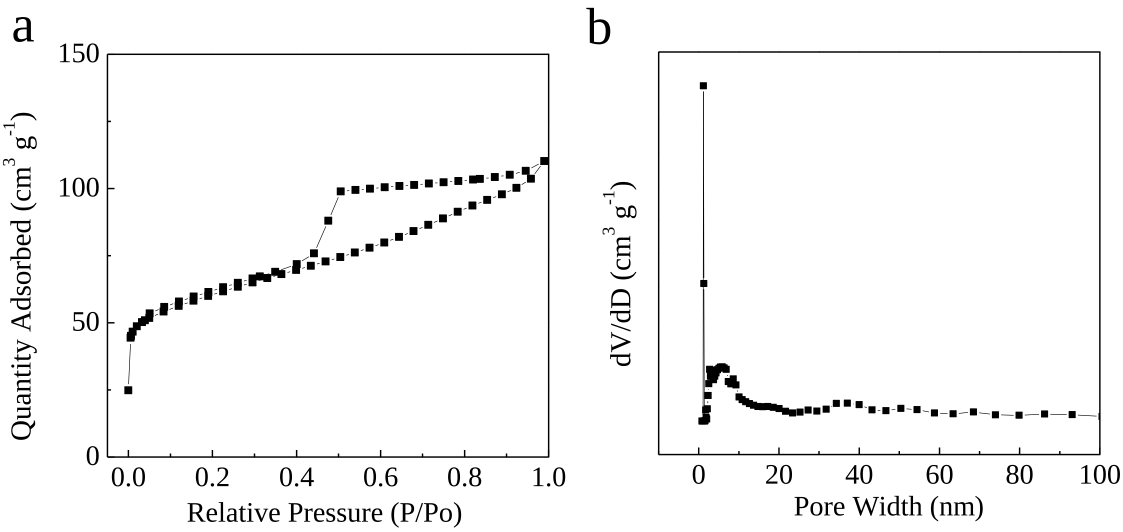

Figure S10. a) N_2 adsorption-desorption isotherms, b) the corresponding pore size distribution curves of Fe_3O_4@NC.

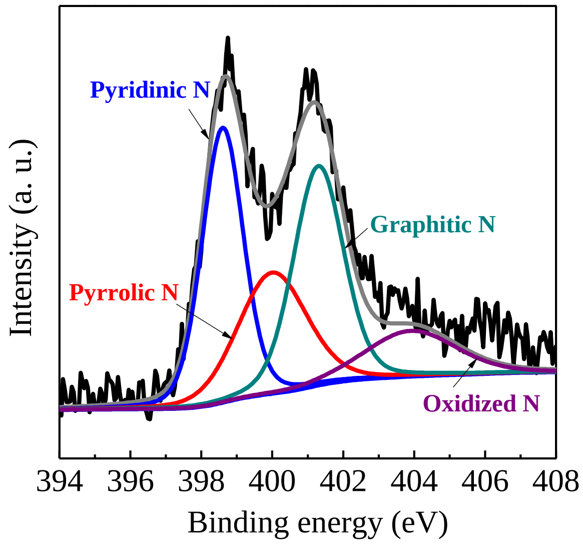

Figure S11. N 1s XPS spectrum of Fe_3O_4@NC.

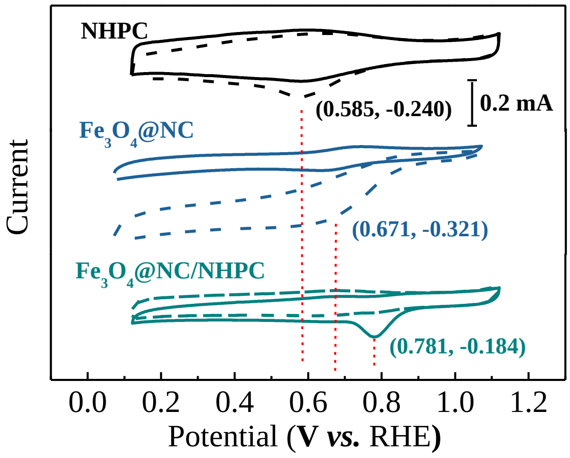

Figure S12. CV curves of NHPC, Fe_3O_4@NC and Fe_3O_4@NC/NHPC in N_2-saturated 0.5 M H_2SO_4 solution (dash line) and O_2-saturated 0.5 M H_2SO_4 solution (solid line).

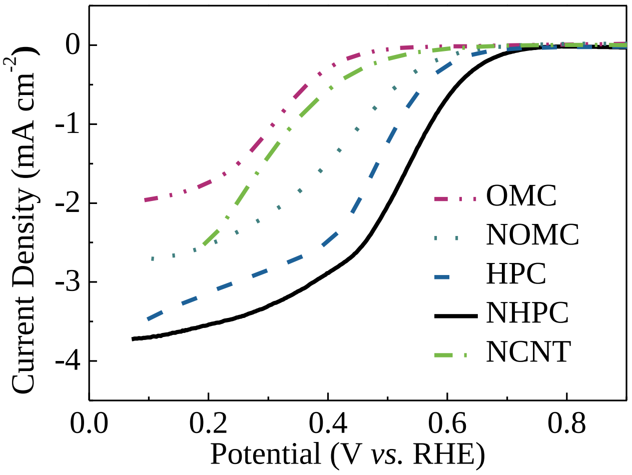

Figure S13. The LSV curves for ordered mesoporous carbon (OMC), nitrogen-doped ordered mesoporous carbon (NOMC), HPC, NHPC in O_2-saturated 0.5 M H_2SO_4 solution. OMC and NOMC were prepared by the hydrothermal reaction in the presence of an ordered mesoporous silica (SAB-15) hard template with sucrose as the carbon precursor.[5]

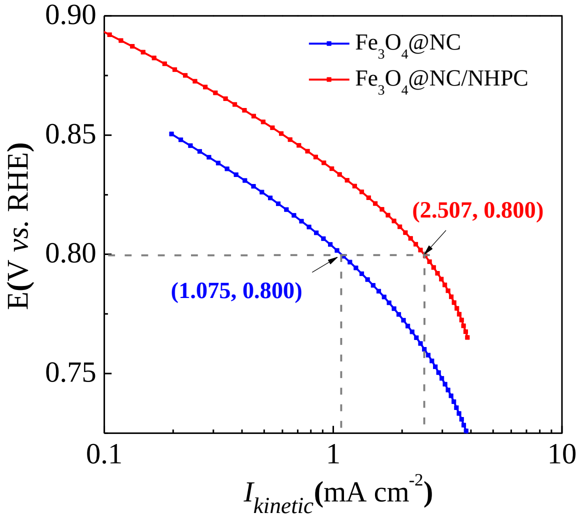

Figure S14. Tafel plots of Fe_3O_4@NC and Fe_3O_4@NC/NHPC obtained from the RDE data.

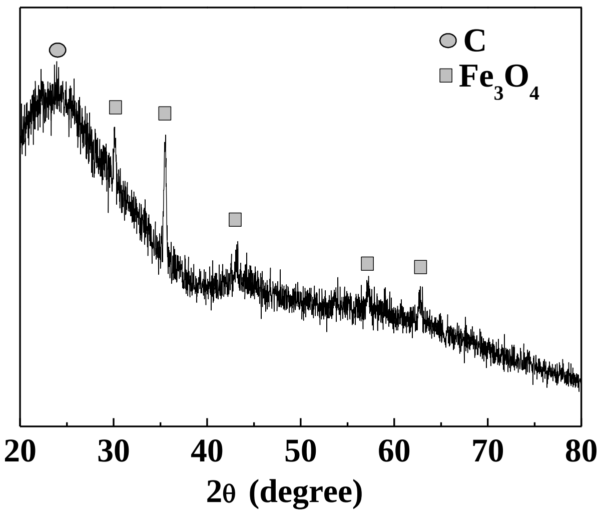

Figure S15. XRD spectrum of Fe_3O_4@C/HPC.

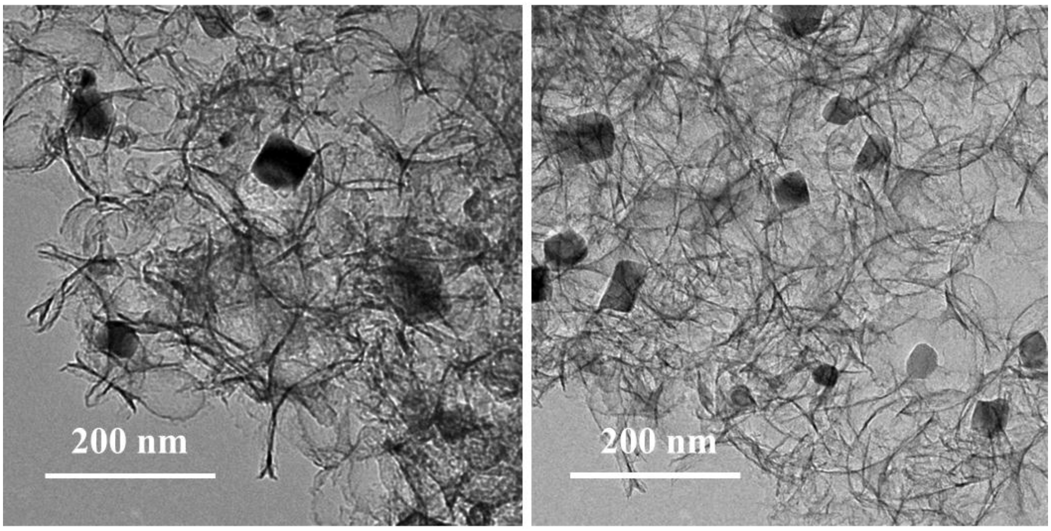

Figure S16. TEM images of Fe_3O_4@C/HPC.

The as-prepared Fe_3O_4@C/HPC showed similar XRD patterns and morphology feature to Fe_3O_4@NC/NHPC.

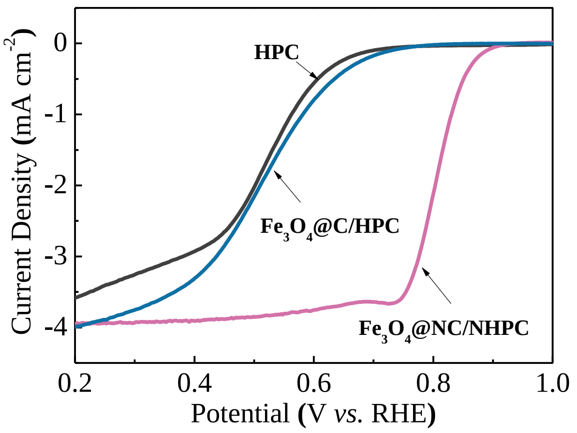

Figure S17. LSV curves for HPC and Fe_3O_4@C/HPC in O_2-saturated 0.5 M H_2SO_4 solution at 1600 rpm.

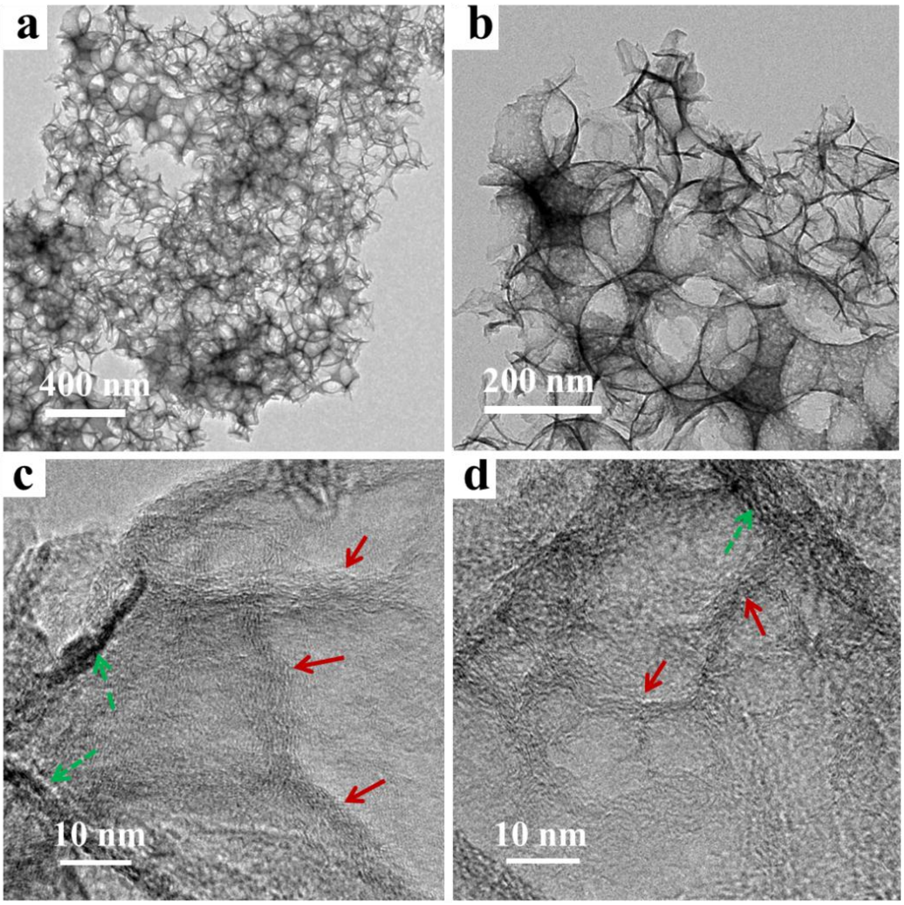

Figure S18. TEM images of Fe_3O_4@NC/NHPC-w.

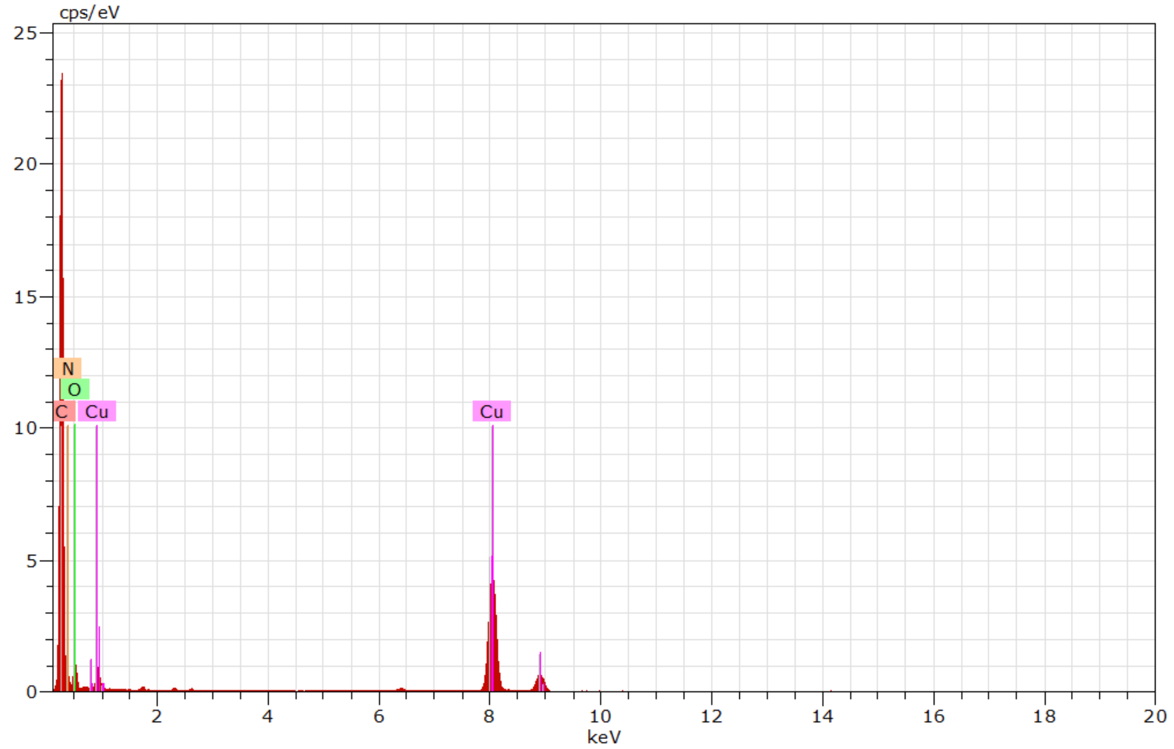

Figure S19. EDS analysis of Fe_3O_4@NC/NHPC-w.

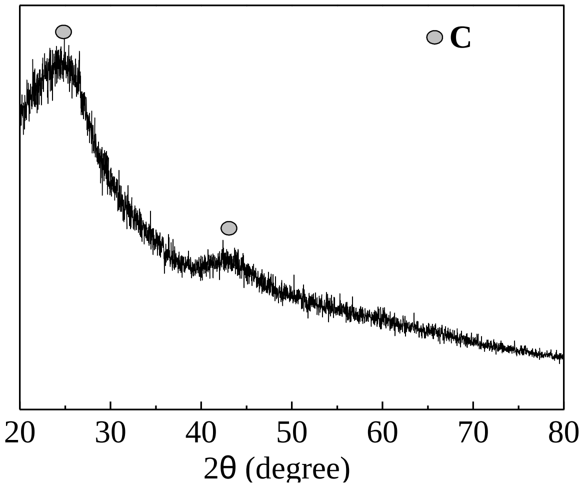

Figure S20. XRD spectrum of Fe_3O_4@NC/NHPC-w.

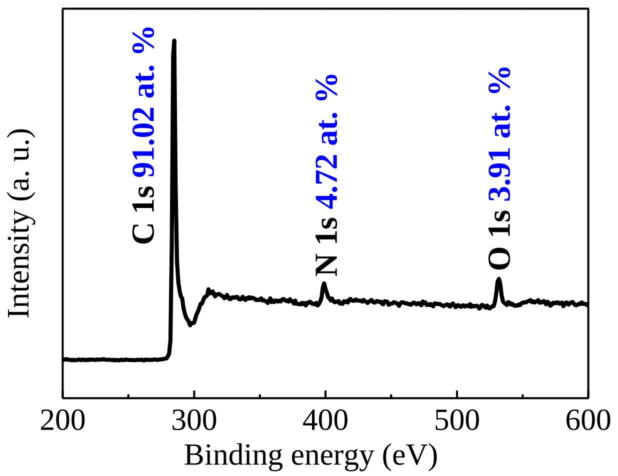

Figure S21. XPS spectrum of NHPC.

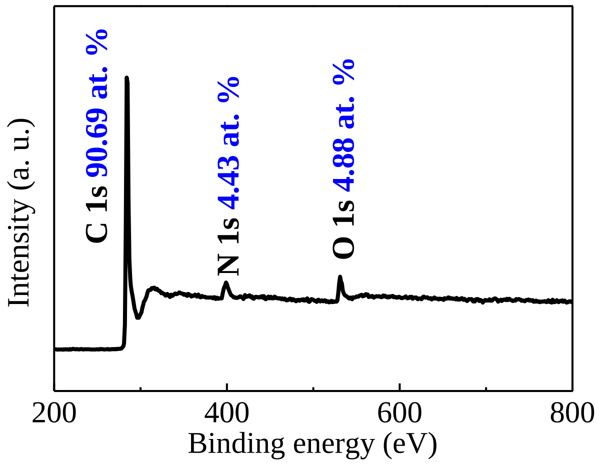

Figure S22. XPS spectrum of Fe_3O_4@NC/NHPC-w.

No metal or metallic species were observed in TEM images over the entire visible range. The corresponding EDS analysis confirmed the inexistence of Fe element. In accordance with TEM and

EDS results, XPS of Fe_3O_4@NC/NHPC-w was free of Fe element, suggesting that Fe species were absolutely washed.

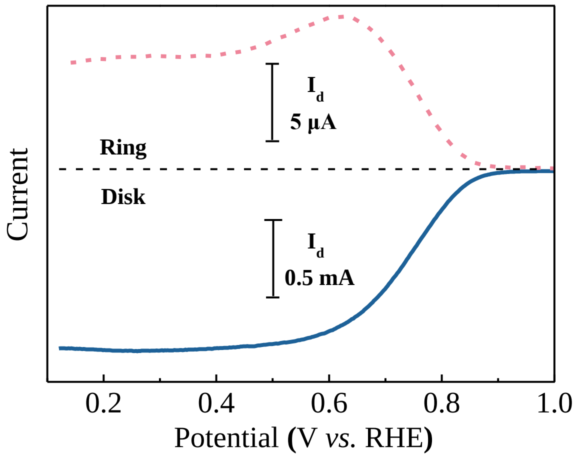

Figure S23. RRDE voltammograms recorded with Fe_3O_4@NC/NHPC in O_2-saturated 0.5 M H_2SO_4 solution at 1 600 r.p.m. The disk potential was scanned at 10 mV s^{-1} and the ring potential was constant at 1.2 V versus SCE.

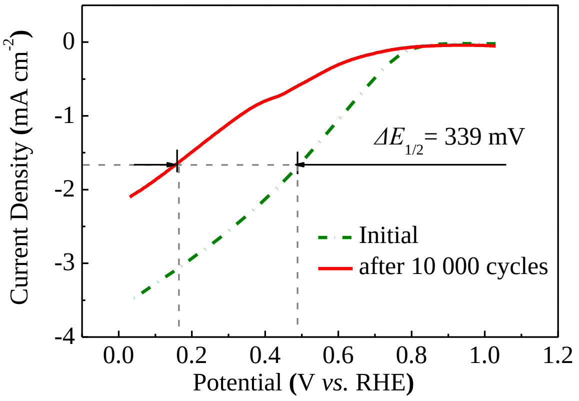

Figure S24. RDE voltammograms of Fe_3O_4@NC catalysts before and after 10 000 cycles in O_2-saturated 0.5 M H_2SO_4 solution.

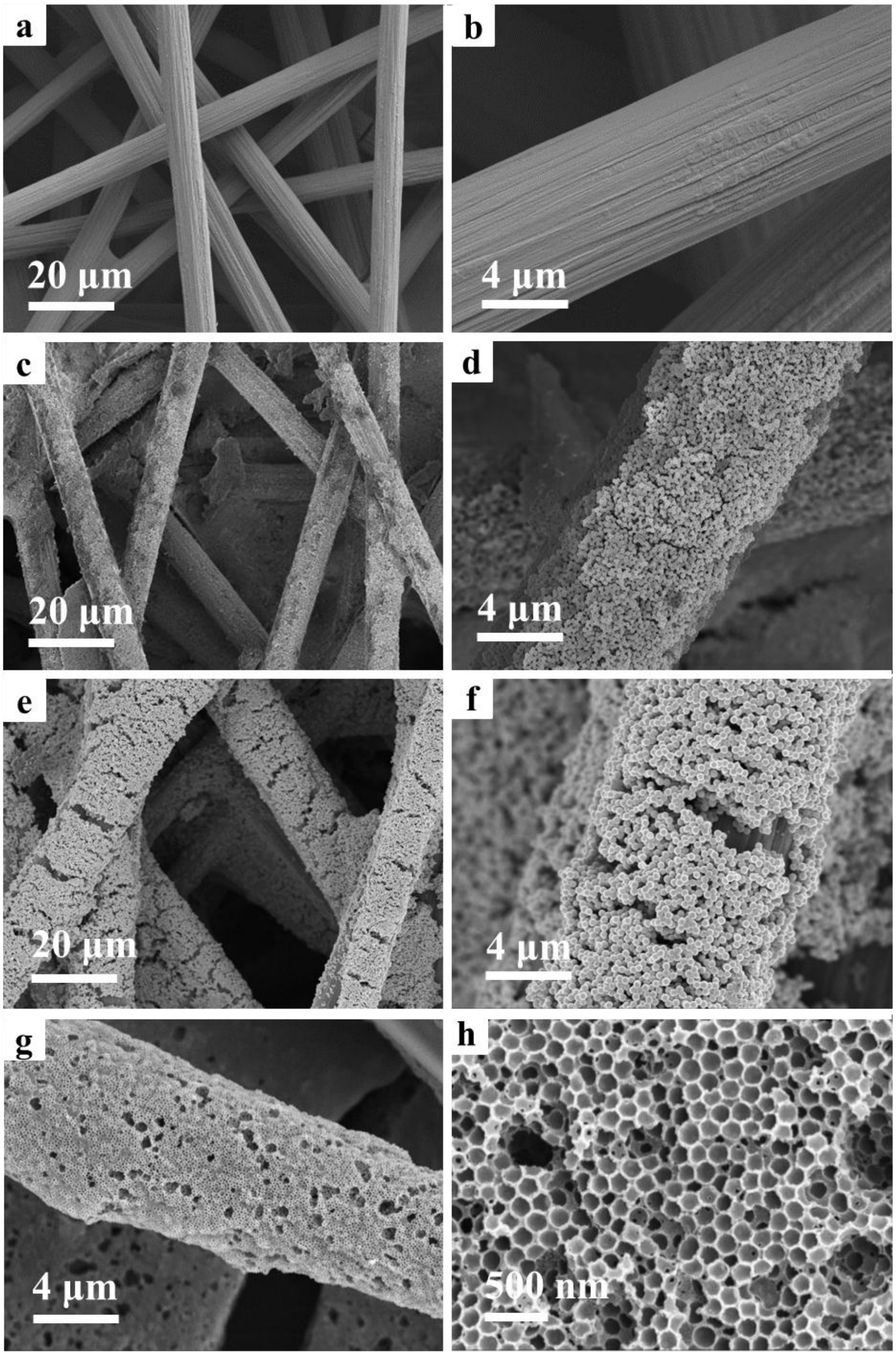

Figure S25. SEM images of a-b) origin CP, c-d) SiO_2/CP, e-f) carbon coated SiO_2/CP, e-f) NHPC/CP.

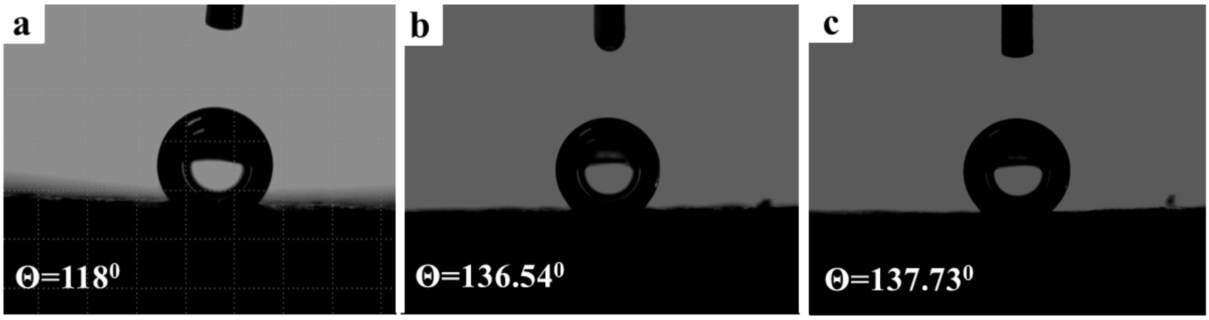

Figure S26. Contact angle measurements of a) original CP, b) Fe_3O_4@NC/NHPC/CP-E and c) Fe_3O_4@NC/NHPC/GDL-S.

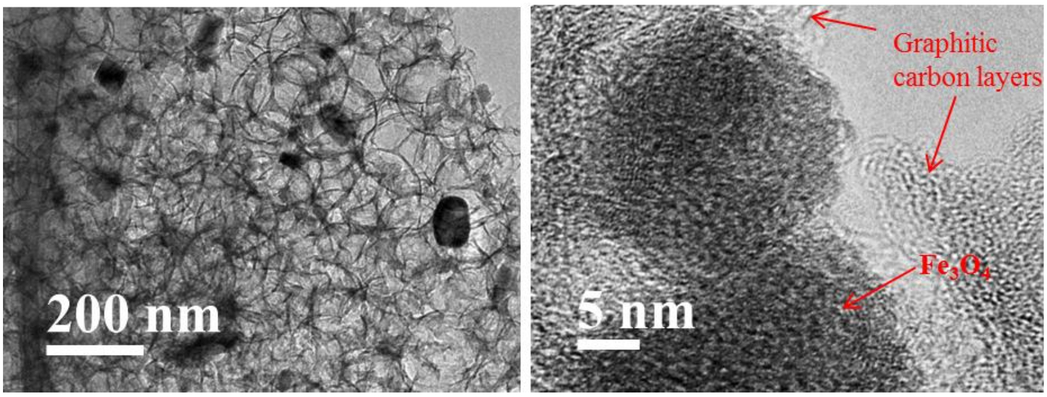

Figure S27. TEM images of Fe_3O_4@NC/NHPC obtained from Fe_3O_4@NC/NHPC/CP-E.

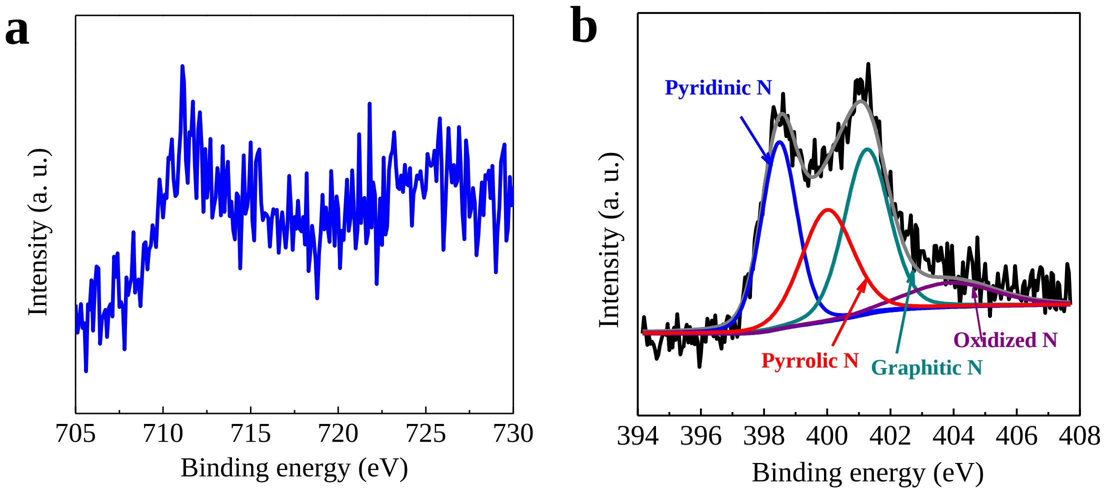

Figure S28. a) Fe 2p XPS spectrum; b) N 1s XPS spectrum of Fe_3O_4@NC/NHPC/CP-E.

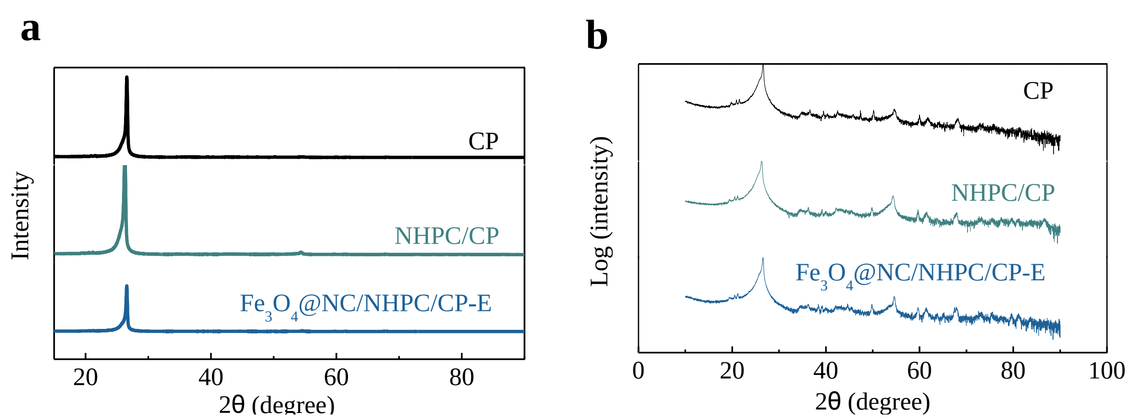

Figure S29. XRD spectrum of CP, NHPC/CP and Fe_3O_4@NC/NHPC/CP-E.

The as-prepared samples exhibited the same XRD spectrum as raw CP, consisting of a major peak at around 25°, a minor peak at around 55° and several weak signals. All of these peaks correspond to high graphitic carbon, coming from the CP itself. The disappearance of Fe_3O_4 might be caused by the strong intensity of CP that affected or even covered up the weak diffraction of Fe_3O_4. The existence of Fe_3O_4 was verified by the XPS analysis.

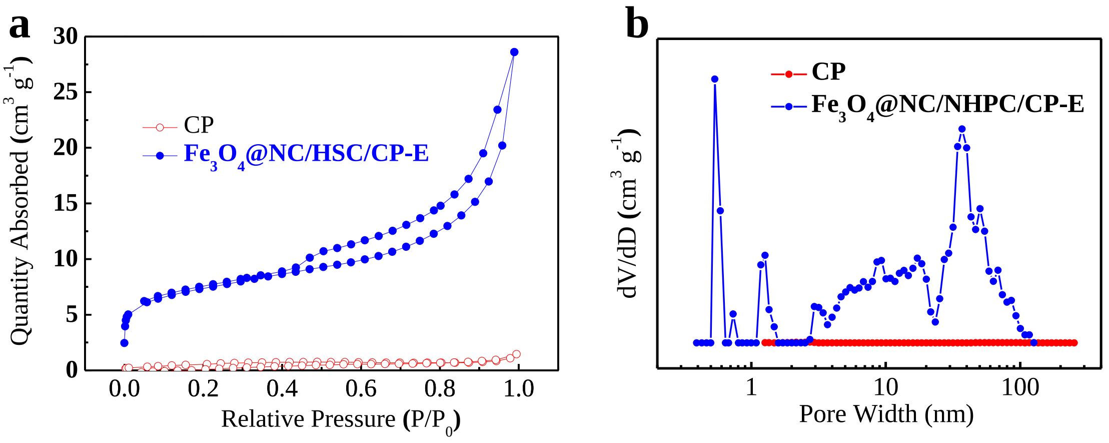

Figure S30. a) N_2 adsorption-desorption isotherms, b) the corresponding pore size distribution curves of CP and Fe_3O_4@NC/NHPC /CP-E.

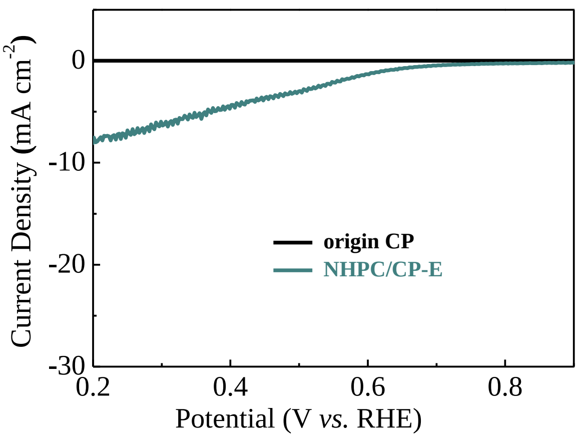

Figure S31. ORR polarization curves of original CP and NHPC/CP-E in O_2-saturated 0.5 M H_2SO_4.

Table S1. Textural properties of Fe_3O_4@NC, NHPC and Fe_3O_4@NC/NHPC.

Samples	BET surface area ($m^2\ g^{-1}$)	Total pore volume ($cm^3\ g^{-1}$)	Pore diameter center (nm)
Fe_3O_4@NC	218	0.15	1.2, 6.3
NHPC	1106	1.74	0.5, 1.2, 34.0
Fe_3O_4@NC/NHPC	768	1.40	1.2, 37.3

Table S2. The surface element distribution of the Fe_3O_4@NC/NHPC, Fe_3O_4@NC, NHPC and Fe_3O_4@NC/NHPC obtained by XPS.

Samples	C content (at. %)	Fe content (at. %)	N content (at. %)	O content (at. %)
Fe_3O_4@NC/NHPC	88.12	5.00	6.35	0.53
Fe_3O_4@NC	87.48	7.37	4.05	1.11
NHPC	91.02	0	4.72	3.91
Fe_3O_4@NC/NHPC-w	90.69	0	4.43	4.88

Table S3. The nitrogen species and relative ration of the Fe_3O_4@NC/NHPC, Fe_3O_4@NC, NHPC and Fe_3O_4@NC/NHPC obtained by XPS.

Samples	Binging energy (eV)	Species	Relative ration (%)
Fe_3O_4@NC/NHPC	398.5	Pyridinc N	41.3
	400.0	Pyrrolic N	20.1
	401.3	Graphitic N	26.2
	403.9	Oxidized N	12.3
Fe_3O_4@NC	398.5	Pyridinc N	32.5
	400.0	Pyrrolic N	23.9

	401.3	Graphitic N	31.6
	403.9	Oxidized N	12.0
NHPC	398.5	Pyridinc N	32.8
	400.0	Pyrrolic N	52.7
	401.3	Graphitic N	14.5
Fe_3O_4@NC/NHPC-w	398.5	Pyridinc N	36.2
	400.0	Pyrrolic N	25.7
	401.3	Graphitic N	31.0
	403.9	Oxidized N	7.0

3. Reference

[1] W. Stöber, A. Fink, *J. Colloid Inter. Sci.* **1968**, *26*, 62.

[2] J. Xu, Z. Wang, D. Xu, L. Zhang, X. Zhang, *Nat. Commun.* **2013**, *4*, 2438.

[3] Y. Liang, Y. Li, H. Wang, J. Zhou, J. Wang, T. Regier, H. Dai, *Nat. Mater.* **2011**, *10*, 780.

[4] a) Z. Li, G. Li, L. Jiang, J. Li, G. Sun, C. Xia, F. Li, *Angew. Chem. Int. Ed.* **2015**, *54*, 1494; b) Y. Li, M. Gong, Y. Liang, J. Feng, J. E. Kim, H. Wang, G. Hong, B. Zhang, H. Dai, *Nat. Commun*. **2013**, *4*, 1805; c) Q. Wang, Z. Zhou, Y. Lai, Y. You, J. Liu, X. Wu, E. Terefe, C. Chen, L. Song, M. Rauf, N. Tian, S. Sun, *J. Am. Chem. Soc.* **2014**, *36*, 10882.

[5] K. Wang, Y. Wang, Z. Liang, Y. Liang, D. Wu, S. Song, P. Tsiakaras, *Appl. Cata. B* **2014**, *147*, 518.
